# The effects of a temporal processing-based auditory training program on the auditory skills of elderly users of hearing aids: a study protocol for a randomized clinical trial

**DOI:** 10.12688/f1000research.22757.2

**Published:** 2020-07-06

**Authors:** Karim Sattari, Nariman Rahbar, Mohsen Ahadi, Hamid Haghani

**Affiliations:** 1 Department of Audiology, Rehabilitation Research Center, School of Rehabilitation Sciences, Iran University of Medical Sciences, Tehran, Iran; 2 Department of Biostatistics, School of Management and Information Technology, Iran University of Medical Sciences, Tehran, Iran

**Keywords:** Auditory training, Temporal processing, Hearing aid, Age-related Hearing loss

## Abstract

**Background**: One of the most important effects of age-related declines in neural processing speed is the impairment of temporal resolution, which leads to difficulty hearing in noisy environments. Since the central auditory system is highly plastic, by designing and implementing a temporal processing-based auditory training program, we can help the elderly improve their listening skills and speech understanding in noisy environments.

**Methods: **In the first phase of this research, based on the theoretical framework of temporal processing, an auditory training solution was developed as a software program. In the second phase, which will be described in the present study, the effects of the designed program on the listening skills of the elderly users of hearing aids (age: 60-75 years) will be studied in the control and intervention groups. In the intervention group, the auditory training program will be implemented for three months (36 sessions), and the results of central tests (GIN, DPT, QuickSIN) and the electrophysiological speech-ABR test will be compared in both groups before, immediately and one month after the intervention.

**Discussion**: Since temporal processing is not sufficient in auditory training programs for the elderly with hearing impairments, implementation of a temporal processing-based auditory training program can reduce hearing problems in noisy environments among elderly users of hearing aids.

**Trial registration: **This study was registered as a clinical trial in the Iranian Registry of Clinical Trials ( IRCT20190921044838N1) on December 25, 2019.

## Introduction

One of the most important issues in today’s world is the phenomenon of aging. With advancing age, the prevalence of chronic diseases increases. Statistics show that at least one chronic disease occurs in 80% of the elderly over 65 years ^1^. According to previous findings, the prevalence of chronic diseases increases up to the age of 75 years. One of the most common problems facing the elderly is hearing loss. Hearing loss is the second most common health problem following arthritis in the elderly over 65 years ^1^.

Age-related hearing loss or presbycusis is a progressive disease, characterized by audiometric threshold shift and impaired speech comprehension, especially in noisy environments ^2, 3^. Many studies have shown reduced speech comprehension in the presence of competitive noise in the elderly, even with normal hearing sensitivity ^4– 8^. Difficulty in speech comprehension in noisy environments has been attributed to a variety of factors, which are not fully understood ^4^. Nonetheless, evidence shows that defects in the peripheral auditory system, retrocochlear processing, and central auditory processing may contribute ^7^.

One of the important factors in speech comprehension in noisy environments is precise neural timing. With advancing age, individuals need more time to respond to sensory input and develop neural slowing. One of the most pronounced outcomes of neural slowing is damage to temporal resolution, which leads to difficulty hearing in noisy environments ^9^. There are many potential biological reasons for the elderly’s slower responses, such as reduced myelin integrity, prolonged nerve recovery, reduced brain connections, and reduced neural synchrony. Perceptual and neurophysiological studies have shown that central auditory processing is also impaired by hearing loss and aging. Among central auditory processing skills, those related to temporal processing are more influenced by age than others ^10^.

Many studies have shown the effects of auditory training on cortical plasticity in animals ^11– 13^ and humans ^14, 15^. Generally, neuroplasticity is not specific to the cortex, which receives input from the brainstem and thalamus ^16^, and some animal studies have indicated plasticity in subcortical structures ^17– 22^. However, there are limited studies on subcortical plasticity among the elderly following auditory training and education ^23^. Music therapy has been used to improve speech perception in the presence of noise in the elderly, and it is believed to be effective in improving the separation of simultaneous speech sounds by enhancing basic frequency and harmonic reception ^24– 27^.

We can modify the physiological representation of sounds using training exercises, as training-dependent physiological changes can lead to improved perception; this phenomenon is sometimes referred to as learning-related plasticity ^28^. In animal studies, it was found that with training, we can change auditory mapping and time decoding. Overall, training-related physiological changes can result in several processes, including an increased number of neurons responsive to the sensory field, increased neuronal synchrony, and neuronal de-correlative processes, associated with inconsistent neuron activity (i.e., each neuron has a specific and distinct function). In other words, with training, neurons in the same category are arranged in a way that they can represent all of the distinct and minor features of the stimulus. In this process, it is assumed that information shared between two stimuli is ignored, but the response to the distinct features of each stimulus is increased ^28^.

Today, modulation of the auditory system using training exercises to enhance perception is one of the most important research trends. Substantial evidence suggests that humans, with or without hearing loss, can increase their ability to perceive spectral and temporal features of acoustic stimuli through auditory training exercises. Auditory training exercises, which are mainly classified into analytical (bottom-up), combined (top-down), and mixed (combination of bottom-up and top-down methods) types, are designed to improve the individual’s ability to understand auditory events through repeated listening exercises ^29^.

Analytical exercises emphasize the acoustic content of the signal (i.e., spectral, temporal, and intensity features), and one’s task is to identify and differentiate different sounds. On the other hand, combined training seeks to improve one’s signal understanding by enhancing attention to stimuli, combining the stimuli, and using contextual information ^29^. Today, most auditory training programs, designed for individuals with communication disorders, use analytical models, which are also considered in the present study. In this study, we aimed to determine the relationship between the temporal characteristics of speech processing, as the basis of speech perception in noisy environments with competitive sounds, and temporal processing-based auditory training.

## Methods

### Ethical statement

The used tests do not cause any complications for the participants, and the participants can withdraw from the study at any stage. All auditory training examinations and sessions are free of charge. Participants with knowledge of objects and details of the research will be included after their written informed consent is obtained by one of the researchers of this study. The nature of the tests and rehabilitation program is such that there are no side effects or harm for participants. The consent form is provided as *Extended data* ^30^. The confidentiality of data will be respected during the study as explained in the consent form. If the results are positive, the auditory training program will be also implemented for the control group. All ethical principles of the Medical Ethics Committee of IUMS will be respected in this study and two anonymous reviewers will be chosen by the deputy of the IUMS research review board to assess the final report of the study. The study does not have an audit and only the final report of the research project will be submitted. The Medical Ethics Committee approved the study protocol (IR.IUMS.REC.1397.652). Researchers will send any amendments to the protocol in the future to the ethics committee.

All data will be entered into forms which are prepared for data collection (see Table 3; *Extended data*) ^30^ and the participant files will be stored at study site and will be maintained in a secure place and manner. Participants’ files will be stored securely after completion of the study and the study data will be available publicly on a repository.

### Study design

This clinical trial protocol is the second part of a larger research project. In the first part, the intervention was developed based on the theoretical framework of temporal processing, which is available as an auditory-based training software program (at home PC-based training program) ^31^. In the second part (simple randomized clinical trial), the effect of the designed training program will be examined. For this purpose, authors will evaluate 60–75 years-old elderly with hearing loss, who live in Tehran and are referred to the Audiology Clinic of the Rehabilitation School of Iran University of Medical Sciences with more than three months of experience in using binaural hearing aids and will be selected based on the inclusion criteria for entering the study. Participants will be invited to take part in the study face-to-face at the clinic.

The participants will be randomly divided into two groups. The intervention group will participate in the auditory training program, while the control group will not be involved in the auditory training program; both groups will be matched for age and gender. For this purpose, random numbers are assigned to individuals based on a random number table. Subjects with odd numbers are assigned to the control group, while those with even numbers are assigned to the intervention group. This process will be carried out by an audiology department employee, who is not involved in the study. Allocation concealment will be performed by random allocation cards using computer-generated random numbers.

### Inclusion criteria

The inclusion criteria are as follows: 1) age range of 60–75 years; 2) mild to moderate bilateral sensorineural hearing loss; 3) mean hearing threshold of 40–55 dBHL (averaged over 0.5, 1, 2, and 4 kHz) in both ears; 4) bilateral hearing loss; 5) use of binaural hearing aids for more than three months; 6) normal middle ear function ^32^; 7) lack of cognitive problems based on the results of Mini-Mental State Examination (MMSE) ^33^; 8) having a high-school diploma or higher; 9) right-handedness based on the Edinburgh Handedness Inventory ^34^; and 10) being a monolingual (Farsi).

### Exclusion criteria

Unwillingness to cooperate with the study at any stage and failure to meet any of the inclusion criteria.

### Study procedures

This research consists of two phases, the first of which has been already carried out.

***Phase 1: Design and development of the temporal processing-based auditory training program.*** The auditory training program was designed based on the theoretical framework of temporal processing and was presented to 10 academic experts to determine its content validity. After that, the program was reviewed by an expert panel, and disagreements were resolved. The experts were academic members of IUMS, Tehran University of Medical Sciences (TUMS), and Shahid Beheshti University of Medical Sciences (SBUMS). This program has been approved by the Medical Ethics Committee of Iran University of Medical Sciences (IR.IUMS.REC.1397.652) and is described in detail below.

The program is based on different aspects of temporal processing consisting of five tasks while one or more aspects are challenged in all tasks, including:

1. Detecting the number of stimuli

This item contains five stimuli (frequencies of 500, 1000, 2000, 4000 Hz and white noise) that are presented in single, binary, or ternary with different combinations for the users and their task is to count the number of stimuli heard. In the first level session, the duration of the stimuli and the gap between the stimuli is 250 ms. In each session, the duration and gap between stimuli is decreased by 5 ms, so in the last session (session 36) the duration and the gap between the stimuli reaches 75 ms.

2. Detecting the pitch of the stimuli

This item contains five stimuli (frequencies of 250, 500, 1000, 2000, 4000 Hz) that are presented in single, binary, or ternary with different combinations for the users and their task is to tell the number of stimuli that are similar in frequency. In the first session, duration of the stimuli and the gap between the stimuli is 1000 ms. In each session, the duration and gap between stimuli decreases by 25 ms, so in the last session (session 36) the duration and the gap between the stimuli reaches 125 ms. The initial sessions use the stimuli that have the highest octave distance (four octaves), while in the final sessions the distance is minimized (one octave).

3. Detecting the duration pattern

This item contains three stimuli (frequencies of 500, 1000, 2000 Hz) and each stimulus is provided to the users with different triple combinations of long or short duration and their task is to determine the number of stimuli that are similar in terms of duration. In the easy stage (1st to 4th week) the long duration is 600 ms and the short duration is 300 ms. However, the gap between the stimuli in the first week is 250 ms, which decreases by 50 ms per week and reduces to 100 ms in the fourth week. The stimulus of the first session of every week is 500 Hz, the second session is 1000 Hz and the third session is 2000 Hz. The same is true for the medium stage (fifth to the eighth week) and the hard stage (9th to 12th week). In the medium stage, the long stimulus duration is 500 ms and the short stimulus duration is 250 ms and at the hard stage the long stimulus duration is 400 ms and the short stimulus duration is 200 ms.

4. Detecting the number of nonsense speech stimuli in noise

This item contains three nonsense speech stimuli (da / ta / ba) with different signal-to-noise ratios for both male and female voices in single, binary or ternary and in different combinations are provided in the presence of background noise and the task is to tell the number of stimuli they have heard. The signal-to-noise ratio starts at 12 dB in the first session, and both sessions decrease by 1 dB (one session for male and one for female), until finally, in the last two sessions, the signal-to-noise ratio decreases to -5 dB.

5. Detecting the gap in noise

The stimulus of this item is white noise, which is provided to clients with a 6-second duration, with zero to three gaps of different durations, and they should determine the number of gaps they have heard. The gap duration starts from 40 - 41 - 42 ms in the first session and each session decreases by 1 ms until session 33, when each session decreases by 0.5 ms until the last session (36) when it has decreased to 7.5 - 8 - 8.5 ms (see Table 1, *Extended data*) ^30^.

The program consists of three stages (easy, medium, and hard), each with 12 levels (sessions). At each level, five temporal processing items are considered with five exercises. Since each exercise should be repeated twice, each auditory training session consists of 50 exercises, which are performed by the subject according to the description of each item. Since the program consists of 36 sessions, the auditory training program comprises 1800 exercises (see Table 2, *Extended data*) ^30^. One or more aspects of temporal processing are challenged in all items. In the first sessions, the user’s task is simple, while in the subsequent sessions, it becomes more difficult, requiring greater precision. Indeed, the program is based on the repetition of exercises.

***Phase 2: Effect of the temporal processing-based auditory training program.*** This phase will be performed in three steps: before, during, and after the training program.

1. Assessments before the auditory training program:

In this phase, the following assessments will be performed by researchers for both the control and intervention groups in the audiology clinic of IUMS:

- The subject’s case history will be taken to examine the inclusion and exclusion criteria.
- Otoscopy and behavioral tests, including pure-tone average (PTA), speech tests such as speech recognition threshold (SRT) and speech discrimination score (SDS), and tympanometry, will be performed.
- MMSE will be applied to assess the absence of cognitive impairment in the participants.
- An electrophysiological test of speech auditory brainstem response (ABR) will be carried out. By studying and comparing the changes in amplitude, latency, and content of frequency responses, the possible effects of the auditory training intervention on the isolation of simultaneous speech sounds will be determined electrophysiologically. Regarding the objective nature of changes in electrophysiological tests, electrophysiological evidence can confirm and demonstrate behavioral changes. Since speech ABR is a suitable test for evaluating subcortical auditory processing mechanisms in speech comprehension in the presence of background noise ^35^, a clear relationship between the stimuli and brainstem responses allows for direct comparisons between frequency and temporal components of the stimulus and response. Previous research has shown a relationship between speech comprehension in the presence of background noise and spectral and temporal components of speech ABR in children ^4^, adults ^36^, and the elderly ^4^. Changes in this test may be an objective reason for altering the function of the nervous system.
- An ABR test will be performed with a speech stimulus, using the Bio-Logic Navigator Pro System. In this test, a non-inverting electrode is placed at Cz, and the inverting electrode is placed on the right ear lobule, and a ground electrode is placed on the forehead. The impedance value of all electrodes is below 5 kΩ (maximum=1.5 kΩ). The stimulus is a 40-msec synthetic /da/ syllable, used in previous studies by the Auditory Neuroscience Laboratory of Nina Kraus *et al.* ^15^ at Northwestern University (USA). The test will be performed in a quiet place with closed eyes (or during sleep) while leaning against a comfortable chair in an acoustic room with low light and low ambient noise. No cognitive stimulus will be used during the experiments ^7^.
- Central tests, including gap detection in noise (GIN), duration pattern test (DPT), and QuickSIN, will be performed. These tests are sensitive to the central auditory nervous system (CANS) lesions but are not affected by peripheral hearing loss ^37^.

**GIN test**: This test consists of a series of 36 different six-second segments of white noise. Each segment contains zero to three gaps of silence. The interval between the noise segments is five seconds, and the duration of silence gaps is 2, 3, 4, 5, 6, 8, 10, 12, 15, and 20 msec. Ten practice items are presented before starting the test. There are four lists available for the test, which allow for a comparison of the tests. Generally, one list is used for each ear. Although the test is run through a single channel, a second channel is also used by the examiner to control and rate the responses. The test includes two criteria for examining the results: 1) a threshold for detecting gaps in noise; and 2) the percentage of correct responses for each ear. The gap detection threshold in noise is considered as the shortest gap duration for which there are four out of six correct responses. The percentage of correct responses is the mean percentage of correct responses during the test ^38^.
**DPT test**: This test consists of three pure tones in each segment. The frequency of each tone is 1000 Hz, and its duration is either 250 msec (short) or 500 msec (long). The gap between the tones is 300 msec. Generally, there are six patterns for this test (LLS, LSL, LSS, SLS, SLS, SLL, and SSL), and a six-second gap of silence separates the segments. The percentage of correct responses is the mean of all percentages of one's correct responses during the test. It is recommended to perform the test at 50 dB SL to the speech recognition threshold or pure tone average (500, 1000, and 2000 Hz). Since intensity has insignificant effects on test performance, it can be also performed at 10 dBSL ^38^.
**QuickSIN test**: In general, QuickSIN is a quick test to quantify one’s ability to hear in the presence of noise. It can be performed using headphones and/or free-field speakers (free-field in this study). In this test, a series of sentences are presented to both ears, and at the same time, a babbling noise is presented to both ears; in other words, noise is competitive with the stimulus. This test is performed at the most comfortable level (MCL). The patient's response is written, and the number of correct repeated words is documented in the “results” sheet. A total of five lists are used for each person.
Each list contains six sentences, and each sentence has five keywords, all of which are presented in babble noise. The noise level is set at six levels (preset on CD) and increases in 5-dB steps; in other words, the test conditions gradually become more and more competitive. The signal-to-noise ratios include 25, 20, 15, 10, 5, and 0 dB. At the end of each list, the total number of correct repeated words is recorded, and the signal-to-noise reduction ratio (SNR loss) is calculated ^32^. ****

2. Implementation of the temporal processing-based auditory training program (only for the intervention group)

Multiple studies have shown that plasticity occurs in the brain 8–12 weeks after training ^39^. Therefore, after the study population is selected, the designed auditory training program will run only for the intervention group over three months. The program will be provided as a DVD to the intervention group for use at home (at home PC-based training program). The auditory training program will be carried out in three easy, medium, and hard stages, with each stage consisting of 12 levels (sessions). Each stage will continue for one month and will be performed by the user for three sessions per week (30–40 minutes per session).

In the designed program, the difficulty of training increases from level 1 to level 36, and the user’s task becomes more difficult requiring more precision. The program is based on the repetition of exercises. In each session, the user is required to earn more than 80% of the scores to pass to the next level. All of the exercises will be performed in several stages over a three-month auditory training period. The exercises start from easy levels in the early days to difficult levels in the final weeks. The program will be implemented at MCL for users with hearing aids in the free field.

To fully familiarize the user with the auditory training program, the first three sessions of the program will be conducted in the audiology clinic in the presence of the researcher to ensure that the user has learned how to use the program correctly and that it is executed properly. Subsequent home sessions will be conducted according to the program protocol and the training of each part of the program. However, in order to retain control and ensure that the program is performed correctly, in all rehabilitation sessions at home, users will be monitored by the researcher via a phone or video call. If a participant is unable to run the program after these tutorials and tips, the training will be discontinued and the person will be removed from the study. ****

3. Assessments after the implementation of the auditory training program

In this stage, central tests, including GIN, DPT, QuickSIN, and speech ABR test, will be repeated for both the control and intervention groups immediately after auditory training, and the outcomes will be compared with the pre-training results. The tests will be repeated after one month to determine the reliability of intervention results to improve the users’ temporal processing.

### Sample size

The sample size is calculated with 80% power and 5% test error (95% confidence interval), based on a similar study by Gil *et al.* ^39^:

$$\text{n}=\frac{\left(z_{1-\alpha /2}+z_{1-\beta }\right)2\left({s_{1}}^{2}+{s_{2}}^{2}\right)}{{\left(\mu_{1}-\mu_{2}\right)}^{2}}$$

According to the abovementioned formula and a 20% dropout rate, the sample size is estimated to be 22 in this study (11 participants per group).

### Data analysis

First, the Chi-square test will be used to examine the normal distribution of each variable. If data distribution is normal, parametric tests, including paired t-test, independent t-test, and ANCOVA test, will be performed. Otherwise, non-parametric Mann-Whitney and Wilcoxon tests will be carried out. Fisher’s exact test will be applied for the homogeneity of the groups. Statistical analysis will be conducted using SPSS version 22.0 (IBM Corporation, New York, USA), and the significance level is considered to be 0.05.

### Study status and dissemination of findings

This trial will start in April 2020 and will be completed in September 2020. To date, enrolment of patients has been performed, and allocation will be done shortly. The trial outcomes will be published in the relevant scientific journals and the results will be communicated to the public, participants and audiologists through a formal report. The results of this study will be communicated to the external funding body through a formal report and there is no limit on the publication of the trial results.

## Discussion

Age-related hearing loss not only influences the physical and emotional activities of the elderly, but also affects their social activities and reduces their quality of life due to various factors, such as depression, social isolation, and reduced self-confidence. One of the important problems in maintaining and promoting the health and quality of life of the elderly is to maintain their independence in daily life activities and to provide suitable conditions for them to continue an active and independent life ^40, 41^. Therefore, by implementing a proper auditory training program, the elderly can comprehend speech in noisy environments; this, in turn, prevents their isolation and reduces the social and financial burdens of their disabilities.

The “plasticity” phenomenon, which has been scientifically approved, suggests the efficacy of auditory training in improving the speech comprehension skills of the elderly. So far, various auditory training methods have been introduced and applied to different studies ^42^. In Iran, we need to design and develop such methods based on the elderly’s needs. Multiple studies have been performed on auditory training for children after receiving hearing aids. However, in Iran, auditory training for elderly users of hearing aids has not been studied, and this is the first research conducted in our country. For this purpose, an elderly auditory training program was designed and implemented with an emphasis on the temporal aspects of speech. Also, regarding the need for special tools and facilities to establish this auditory training program, the proposed program can be used in audiology clinics and academic centers to improve the speech comprehension of elderly users of hearing aids, especially in the presence of background noise.

## Data availability

### Underlying data

No underlying data are associated with this article.

### Extended data

Open Science Framework: The effects of a temporal processing-based auditory training program on the auditory skills of elderly users of hearing aids: a study protocol for a randomized clinical trial. https://doi.org/10.17605/OSF.IO/SB934 ^30^

This study contains the following extended data:

- Consent form for participants of the study.docx
- Table 1 (Details and steps of the rehabilitation program).xlsx
- Table 2 (Schedule of temporal processing-based program).docx
- Table 3 (The data collection sheet of the study).docx
- Table 4 (Participant timeline).docx

### Reporting guidelines

Open Science Framework: SPIRIT checklist for 'The effects of a temporal processing-based auditory training program on the auditory skills of elderly users of hearing aids: a study protocol for a randomized clinical trial': https://doi.org/10.17605/OSF.IO/QPJDK ^30^.

Data are available under the terms of the Creative Commons Attribution 4.0 International license (CC-BY 4.0).

## Software availability

Source code available from: https://github.com/karimsattari/source-code-of-auditory-training/tree/V2.0.0

Archived source code at time of publication: https://doi.org/10.5281/zenodo.3816735 ^31^

License: MIT
