# Supplementary material for: The effects of a temporal processing-based auditory training program on the auditory skills of elderly users of hearing aids: a study protocol for a randomized clinical trial
Source: F1000Res. 2020 Jul 6;9:425. Originally published 2020 May 22. [Version 2] doi: 10.12688/f1000research.22757.2 (PMC7308962; doi:10.12688/f1000research.22757.2)
Supplement: Peer review history

# Peer review: Reviewer response for version 2

**DOI:** 10.5256/f1000research.27424.r66479

**Published:** 2020-07-13

**Authors:** Amineh Koravand

**Affiliations:** 1 School of Rehabilitation Sciences, Audiology and Speech Pathology Department, University of Ottawa, Ottawa, ON, Canada

In this new version, the order of the authors was changed.

Is the study design appropriate for the research question?

Partly

Is the rationale for, and objectives of, the study clearly described?

Yes

Are sufficient details of the methods provided to allow replication by others?

Yes

Are the datasets clearly presented in a useable and accessible format?

Yes

Reviewer Expertise:

Neuro-audiology, speech perception, central auditory processing, enriched auditory experiences, cortical and subcortical auditory evoked potentials.

I confirm that I have read this submission and believe that I have an appropriate level of expertise to confirm that it is of an acceptable scientific standard, however I have significant reservations, as outlined above.

# Peer review: Reviewer response for version 2

**DOI:** 10.5256/f1000research.27424.r66480

**Published:** 2020-07-07

**Authors:** Helen Henshaw

**Affiliations:** 1 National Institute of Health, Nottingham, UK; 2 Hearing Sciences, Division of Clinical Neuroscience, University of Nottingham, Nottingham, UK

In the abstract, the authors should change ‘reduce hearing problems’ to ‘reduce *the impact* of hearing problems’

 The authors did not address these issues highlighted in my first review:

  

- Justification should be offered for each of the inclusion criteria listed.
    
- Justification should be offered for the limited age range of eligible participants (60-75 years old).
- Please state your criteria for pass/fail on the MMSE.

The adaptive paradigm for the training task is not clear from the following text ‘In the designed program, the difficulty of training increases from level 1 to level 36, and the user’s task becomes more difficult requiring more precision’. Is it an adaptive staircase, with what step size across which parameter(s)?

 The authors failed to address any of the following issues highlighted in my first review:

- Similarly, will the training log user input and performance in real-time? On the local PC? How/will these data be accessed and used by the research team? Or is on-task training performance not gathered/assessed?
- If participants are removed from the study, how will this be dealt with (i.e. will they be replaced?)
- Do the authors intend to conduct per-protocol or intention-to-treat analyses? The data analysis section is limited and would benefit from the consideration of multivariate techniques aligned to this type of research (multiple outcome assessments over time).
- The authors should state the outcome data on which the power calculation is based, particularly given that the proposed group size is very small in comparison to other computer-based auditory training intervention studies (see e.g. Henshaw & Ferguson, 2013 for a review) ^1^. Notably, the sample size in Gil (which the authors use as the basis for their calculation) was 'doubled' by including participant data for right and left ears independently (not recommended). I would therefore strongly urge the authors to revisit their power calculation based on pilot data and/or a high quality published study using aligned outcome measures in a similar population.

Although all points warrant action, the final point is a very important issue to address.

 I do not recommend publication of this manuscript in its current form.

Is the study design appropriate for the research question?

Yes

Is the rationale for, and objectives of, the study clearly described?

Yes

Are sufficient details of the methods provided to allow replication by others?

Yes

Are the datasets clearly presented in a useable and accessible format?

Yes

Reviewer Expertise:

Adult aural rehabilitation, auditory training, cognitive training, health behaviour change, cognition, coproduction

I confirm that I have read this submission and believe that I have an appropriate level of expertise to state that I do not consider it to be of an acceptable scientific standard, for reasons outlined above.

## References

[1] : Efficacy of individual computer-based auditory training for people with hearing loss: a systematic review of the evidence. *PLoS One*.2013;8(5) : 10.1371/journal.pone.0062836 e62836 10.1371/journal.pone.0062836 23675431PMC3651281

# Peer review: Reviewer response for version 1

**DOI:** 10.5256/f1000research.25127.r63905

**Published:** 2020-06-30

**Authors:** Helen Henshaw

**Affiliations:** 1 National Institute of Health, Nottingham, UK; 2 Hearing Sciences, Division of Clinical Neuroscience, University of Nottingham, Nottingham, UK

The abstract discussion section is a little unclear and could be misinterpreted as it currently stands. Suggest rewording the sentence 'Since temporal processing is not sufficient in auditory training programs for the elderly with hearing impairments' for clarity.

 Justification should be offered for each of the inclusion criteria listed.

 Justification should be offered for the limited age range of eligible participants (60-75 years old).

 Please state your criteria for pass/fail on the MMSE.

 The authors should add more detail in terms of how trainees progress through the training difficulty levels on the training program. Given that the training is DVD-based, is an adaptive algorithm based on trainee performance possible? Please describe in detail how transitions between difficulty levels will be achieved and managed.

 Similarly, will the training log user input and performance in real-time? On the local PC? How/will these data be accessed and used by the research team? Or is on-task training performance not gathered/assessed?

 If participants are removed from the study, how will this be dealt with (i.e. will they be replaced?)

 Do the authors intend to conduct per-protocol or intention-to-treat analyses? The data analysis section is limited and would benefit from the consideration of multivariate techniques aligned to this type of research (multiple outcome assessments over time).

 The authors should state the outcome data on which the power calculation is based, particularly given that the proposed group size is very small in comparison to other computer-based auditory training intervention studies (see e.g. Henshaw & Ferguson, 2013 for a review) ^1^. Notably, the sample size in Gil (which the authors use as the basis for their calculation) was 'doubled' by including participant data for right and left ears independently (not recommended). I would therefore strongly urge the authors to revisit their power calculation based on pilot data and/or a high quality published study using aligned outcome measures in a similar population.

 I commend the authors on their open study documentation, source code, and SPIRIT checklist.

Is the study design appropriate for the research question?

Yes

Is the rationale for, and objectives of, the study clearly described?

Yes

Are sufficient details of the methods provided to allow replication by others?

Yes

Are the datasets clearly presented in a useable and accessible format?

Yes

Reviewer Expertise:

Adult aural rehabilitation, auditory training, cognitive training, health behaviour change, cognition, coproduction

I confirm that I have read this submission and believe that I have an appropriate level of expertise to confirm that it is of an acceptable scientific standard, however I have significant reservations, as outlined above.

## References

[1] : Efficacy of individual computer-based auditory training for people with hearing loss: a systematic review of the evidence. *PLoS One*.2013;8(5) : 10.1371/journal.pone.0062836 e62836 10.1371/journal.pone.0062836 23675431PMC3651281

# Peer review: Reviewer response for version 1

**DOI:** 10.5256/f1000research.25127.r63904

**Published:** 2020-06-16

**Authors:** Amineh Koravand

**Affiliations:** 1 School of Rehabilitation Sciences, Audiology and Speech Pathology Department, University of Ottawa, Ottawa, ON, Canada

It is a well-written study protocol. The need to have an auditory training program designed for the elderly is critical and very important. The authors are addressing this need by developing a special auditory software. I approve the protocol; however, I do have some comments for improving the study. Most of them are minor and can be handled easily. My two main concerns (explained below under the method section) are related to the participants ‘age and adding a speech perception test at the beginning of the experiment (as a screening tool for measuring the participant's qualification). 

**Introduction:** 

1. The introduction is short, but it may be related to the Journal specification for the study protocol. 
2. Some concept needs to be explained briefly: neural slowing, plasticity, the theoretical framework of temporal processing’, speech perception difficulties, how were these difficulties were measured in the past
3. Some references need to be added. The authors advised to re-read the introduction and add the appropriate references. I am reporting several here, however, the authors need to check the introduction again: 

**For example:** 

 The first paragraph of the introduction (the first 2 sentences): ‘One of the most important issues in today’s world is the phenomenon of aging’. ‘With advancing age, the prevalence of chronic diseases increases’. 

 Third Paragraph: ‘There are many potential biological reasons for the elderly’s slower responses, such as reduced myelin integrity, prolonged nerve recovery, reduced brain connections, and reduced neural synchrony’ and the next sentence: ‘Perceptual and neurophysiological studies have shown that central auditory processing is also impaired by hearing loss and aging.’ 

 Sex paragraph: 'Substantial evidence suggests that humans, with or without hearing loss, can increase their ability to perceive spectral and temporal features of acoustic stimuli through auditory training exercises.'

**Objective/Aim**

 The last paragraph of the introduction is related to the objective. This needs to be re-written. The main objective of the project needs to be presented more clearly.

 Can the authors formulate one or two hypotheses for their project?

**Method section:**

 Inclusion criteria:

 Numbers 3 and 4 can be combined.

 Number 5: why 3 months, based on which studies.

 It will be highly necessary to include one speech perception test (in silence and in noise) for selecting the participant. In fact, not all the participants would have an important speech understanding difficulty in noise. Can the speech perception be measured before enrolling in the project? Since the authors will run a basic audiological evaluation to determine the hearing loss degree, adding a speech test (even a screening one), will be so beneficial.

**Participant's age:**

 I am a little concerned about the participant’s age range, it is an important factor. A 60 years old participant will not have the same auditory capacity (Peripheral and central) compared to a 75 yrs old. Do you have any distribution limits? I mean how would the authors control this potential situation: 10 out of 12 participants are 60 yrs and only 2 are 75 years in the experimental group. I understand that we cannot control all the variables, however, can we concentrate on 70 to 75 yrs population instead of having this large age window (especially for the elderly that each year would bring some kind of declines in their capacity)

**Testing the Auditory training’ effect:**

 Before, immediately and one month after. 

 Can we evaluate the performance three months later as well to be sure about the long-term effect?

 Authors mentioned that the training sessions will be monitored via phone or video call, is it for each session?

 How the authors can be sure that the participants will be practicing 30 to 40 minutes per session (not more or less), is there any way to detect the duration of the practice.

**Discussion:** 

 Authors mentioned that age-related hearing loss not only influences the physical and emotional activities of the elderly but also affects their social activities and reduces their quality of life. Is it possible to add a questionnaire evaluating some of the emotional, social, and/or daily communication patterns?

Is the study design appropriate for the research question?

Partly

Is the rationale for, and objectives of, the study clearly described?

Yes

Are sufficient details of the methods provided to allow replication by others?

Yes

Are the datasets clearly presented in a useable and accessible format?

Yes

Reviewer Expertise:

Neuro-audiology, speech perception, central auditory processing, enriched auditory experiences, cortical and subcortical auditory evoked potentials.

I confirm that I have read this submission and believe that I have an appropriate level of expertise to confirm that it is of an acceptable scientific standard, however I have significant reservations, as outlined above.

# Peer review: Reviewer response for version 1

**DOI:** 10.5256/f1000research.25127.r64063

**Published:** 2020-06-15

**Authors:** Maria Cecília Martinelli Iório

**Affiliations:** 1 Federal University of São Paulo (UNIFESP), São Paulo, Brazil

The abstract is adequately presented. The introduction justifies the completion of the study and the methods provided allow the replication by others.

 The study is important in the area of knowledge of audiology, especially because from this knowledge the indication of rehabilitation can be better conducted.

 Bibliographic references must be updated. I understand that the best reference to justify the prevalence of hearing loss in the elderly is the global burden of disease. Other references must be updated too.

 Vos, T., Abajobir, A. A., Abate, K. H., Abbafati, C., Abbas, K. M., Abd-Allah, F.,  Abera, S. F. (2017). Global, regional, and national incidence, prevalence, and years lived with disability for 328 diseases and injuries for 195 countries, 1990–2016: a systematic analysis for the Global Burden of Disease Study 2016. The Lancet,390(10100),1211-1259.doi:https://doi.org/10.1016/S01406736(17)32154-2 ^1^

 As the text explains age-related hearing loss (ARHL) not only influences the physical and emotional activities of the elderly, but also affects their social activities and reduces their quality of life due to various factors, such as depression, social isolation, and reduced self-confidence. One of the important problems in maintaining and promoting the health and quality of life of the elderly is to maintain their independence in daily life activities and to provide suitable conditions for them to continue an active and independent life.

 I think that the application of questionnaires that evaluate the limitations and restrictions in the activities of daily life is of great importance.

 Tests in controlled situations that do not represent daily activities may not show the improvement targeted by the training.

 Therefore, I believe that it is very important to apply questionnaires that assess the quality of life and the limitations and restrictions in activities of daily living before and after auditory training.

 If it is not possible to include this type of evaluation, consider these aspects when discussing the results and the possible applicability of the proposed training.

Is the study design appropriate for the research question?

Partly

Is the rationale for, and objectives of, the study clearly described?

Yes

Are sufficient details of the methods provided to allow replication by others?

Yes

Are the datasets clearly presented in a useable and accessible format?

Yes

Reviewer Expertise:

Cognitive Hearing

I confirm that I have read this submission and believe that I have an appropriate level of expertise to confirm that it is of an acceptable scientific standard.

## References

[1] : Global, regional, and national incidence, prevalence, and years lived with disability for 328 diseases and injuries for 195 countries, 1990–2016: a systematic analysis for the Global Burden of Disease Study 2016. *The Lancet*.2017;390(10100) : 10.1016/S0140-6736(17)32154-2 1211-1259 10.1016/S0140-6736(17)32154-2 PMC560550928919117

# Peer review: Reviewer response for version 1

**DOI:** 10.5256/f1000research.25127.r63907

**Published:** 2020-06-01

**Authors:** Ahmadreza Nazeri

**Affiliations:** 1 Department of Audiology, School of Rehabilitation, Shahid Beheshti University of Medical Sciences, Tehran, Iran

This study protocol design tries to introduce an innovative temporal processing training software, which will be able to improve temporal processing abilities in the elderly population.

 A complete test battery will be able to show the possible improvements of the above mentioned temporal processing training program in a hearing-impaired elderly population.

 I have a recommendation for you, it would be better to draw a flowchart and show all the details inside it; however, it is only a recommendation

Is the study design appropriate for the research question?

Yes

Is the rationale for, and objectives of, the study clearly described?

Yes

Are sufficient details of the methods provided to allow replication by others?

Yes

Are the datasets clearly presented in a useable and accessible format?

Yes

Reviewer Expertise:

hearing science, amplification, Electrophysiology

I confirm that I have read this submission and believe that I have an appropriate level of expertise to confirm that it is of an acceptable scientific standard.
